# An Unusual Case of Myopericarditis in a Young Woman

**DOI:** 10.7759/cureus.32542

**Published:** 2022-12-15

**Authors:** Inês Figueiredo, Inna Kozyar, Cristina Duarte, Francisco Guimarães

**Affiliations:** 1 Internal Medicine, Hospital da Cuf Descobertas, Lisbon, PRT

**Keywords:** typical angor, myopericarditis, systemic lupus erythematosus, sjögren's syndrome, coxiella burnetti infection

## Abstract

A 48-year-old woman with no history of cardiovascular risk factors was admitted to the emergency room with complaints of angor, dyspnea, and fever in the last 24 h. She was referred for xerostomia and xerophthalmia since 2015. At examination, the patient was polypneic with bibasal crackles. Blood tests showed leukocytosis and increased high sensitivity troponin I and C-reactive protein (CRP). The echocardiogram revealed a small pericardial effusion.

She was diagnosed with myopericarditis and started acetylsalicylic acid (ASA) and colchicine. Laboratory tests indicated the presence of positive antinuclear antibodies, double-strain DNA antibodies (anti-dsDNA), anti-Sjögren's-syndrome-related antigen A (anti-SSA), and lupus anticoagulant antibodies. Positivity for phase II immunoglobulin M and G for *Coxiella burnetti* was detected. As it fulfills the diagnostic criteria for a possible flare of systemic lupus erythematosus (SLE) and SS (triggered by a possible infection by *C. burnetti*) the patient started immunosuppressive therapy. A complete resolution of symptoms with normalization of CRP and troponin I values were observed.

## Introduction

Myopericarditis is a common differential diagnosis of angor in young adults. The main causes of myopericarditis are viral infections (cytomegalovirus, Epstein-Barr virus, herpes virus, enterovirus, parvovirus, hepatitis B and C virus, and human immunodeficiency virus), bacterial infections (*Borrelia burgdorferi*, *Coxiella burnetti*), and autoimmune diseases (SLE, systemic lupus erythematosus and RA, rheumatoid arthritis) [1]. This should be our guiding line in the search for the etiology of this clinical report. 

Although not being a common etiology of myopericarditis in female patients without evident cardiovascular risk factors, autoimmune diseases need to be considered as a possible diagnosis. In 2019 Pasoto et al. [2] described that the prevalence of SLE has increased worldwide, with significant geographic differences. In Europe, prevalence reaches values <40 per 100 000 inhabitants in contrast to >160 per 100 000 inhabitants in some American states [2]. 

Systemic lupus erythematosus first flare can have diverse presentations which justifies the importance of reporting less common cases of inaugural manifestation. In this acute phase of SLE, we have more often positivity for antinuclear antibodies (ANA; which has a sensitivity of 95% and 20% specificity) and anti-dsDNA (with a specificity of 96%) [3]. The institution of early therapy to prevent irreversible damage to the target organ is even more important. 

Even so, since the cause of myopericarditis is an infection (viral or bacterial), the search for an infectious focus and isolation of pathogenic microoganism should be of paramount importance. Because it is a mandatory intracellular proteobacterium, *C. burnetti* is rarely found in cultural exams, but polymerase chain reaction (PCR) serology when positive for immunoglobulin G (IgG) and/or immunoglobulin M (IgM) phase II points to acute infection. **Jacobson and Sutthiwan (2019) [4] conducted a review where 60% of patients with Q fever are asymptomatic, but the remaining 40% appear with fever, myalgia, skin rash, and more commonly with myocardial involvement (myocarditis in 0.6%-0.8% of Q fever). 

The relationship between Q fever and the manifestation of SLE flare is not yet well established, but this case corroborates the idea that there is in fact a possible cause-effect relationship between these pathologies, with some reports in the literature of clinical cases in which both manifested in an overlapping presentation. 

We report a case of rare complexity of a pre-menopausal female patient who presents with symptoms suggestive of myopericarditis without relevant personal history, usual outpatient medication, consumption of toxic substances and without an epidemiological context that justifies a possible zoonosis. It was concluded that the origin of this diagnosis was infection by *C. burnetti* but its role, in this case, was the big question: *C. burnetti* could be the primary cause for myopericarditis but on the other hand could have motivated the appearance of the inaugural flare of SLE and Sjögren's syndrome (SS).

## Case presentation

A 48-year-old woman, living in an urban area, without contact with animals or recent trips, worked at home during the three weeks before coming to the emergency room. 

She referred to a clinical history of bronchial asthma and allergic rhinitis treated with bilastine, montelukast, and terbutaline as a rescue medication. She also referred to a history of xerophthalmia (with the need for daily use of artificial tears), xerostomia, sporadic malar, and peri-orbital eczema with more than five years of evolution that she never valued so she never sought medical help. She denied smoking, ethanolic habits, or consumption of toxic substances. 

The patient went to the emergency room for an episode of lypothymia, oppressive retrosternal pain with irradiation to the cervical region and left shoulder, and tiredness for small efforts with two days of evolution, associated with fever and dyspnea. 

On admission, she presented with a Glasgow Coma Score 15, collaborative and oriented, polypneic in room air with 98% peripheral oxygen saturation, with a rhythmic cardiac auscultation and controlled heart rate. Pulmonary auscultation showed few crackles in both lung bases. No edema of the lower limbs was detected. No other changes to highlight in the objective exam.

Relevant results from complementary exams on admission are normochromic normocytic anemia with hemoglobin of 11.7 g/dL, leukocytosis of 19 100 leukocytes with neutrophilia of 84.4%, C-reactive protein (CRP) 24.7 mg/dL, increased D-dimer of 5776 ng/mL (reference value <500 ng/mL), elevated transaminases (aspartate aminotransferase - AST 135 U/L, alanine transaminase - ALT 183 U/L; considering reference values of 40 U/L for AST and 41 U/L for ALT) and hepatic cholestasis parameters, highly sensitive troponin I with a value of 219 ng/L (reference value <19 ng/L in females), urine II and urinary sediment unchanged, negative severe acute respiratory syndrome coronavirus 2 (SARS-Cov2) test; urine culture and blood cultures also turned out to be negative. 

**Figure 1:**
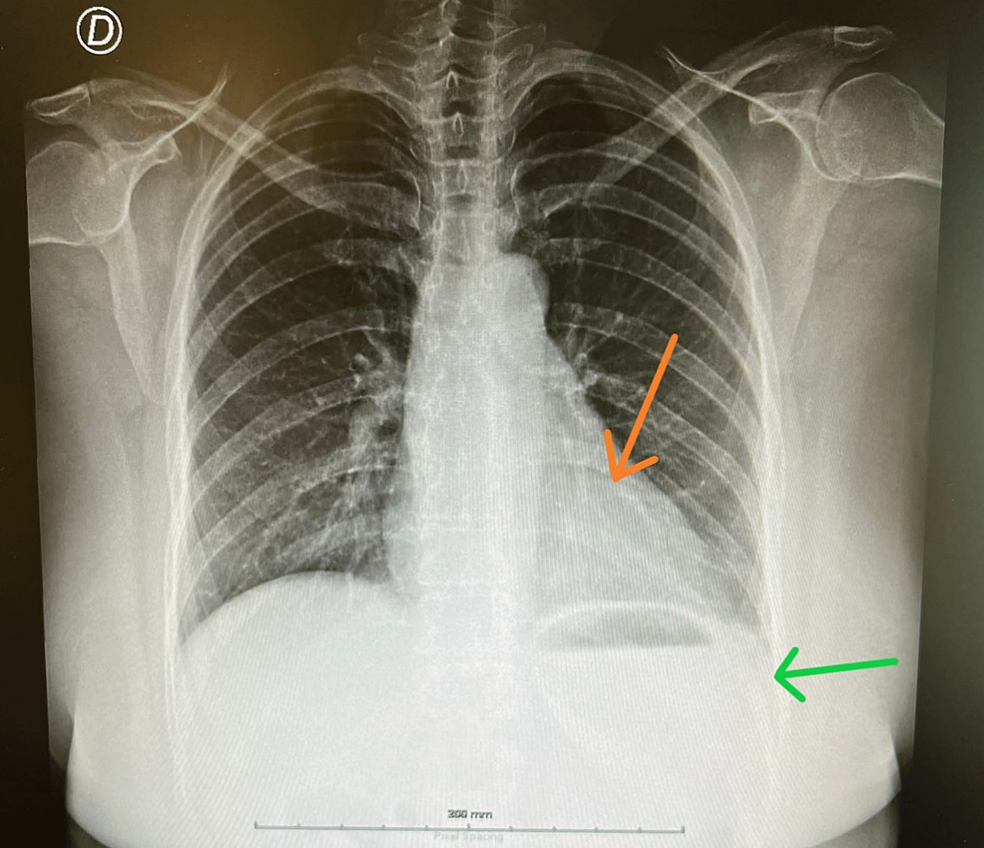
Chest X-ray. Orange arrow indicates a slight increase in cardiothoracic index; Green arrow represents a small pleural effusion on the left thorax.

Chest X-ray showed a slight increase in the cardiothoracic index and a small pleural effusion on the left thorax (Figure 1). Electrocardiogram in sinus rhythm with doubtful ST elevation in DII and aVF and nonspecific changes in repolarization. 

**Figure 2:**
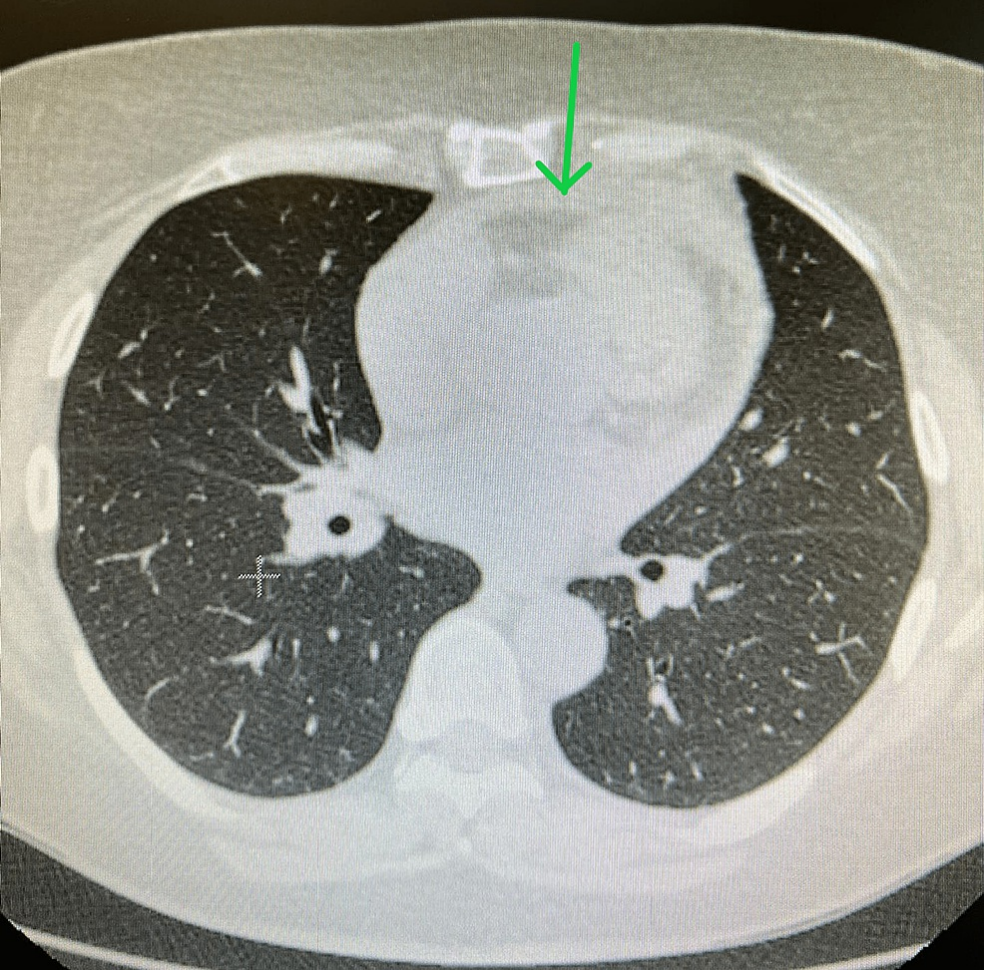
Chest angio-tomography with pericardial effusion. Green arrow indicates a slight pericardial effusion.

**Figure 3:**
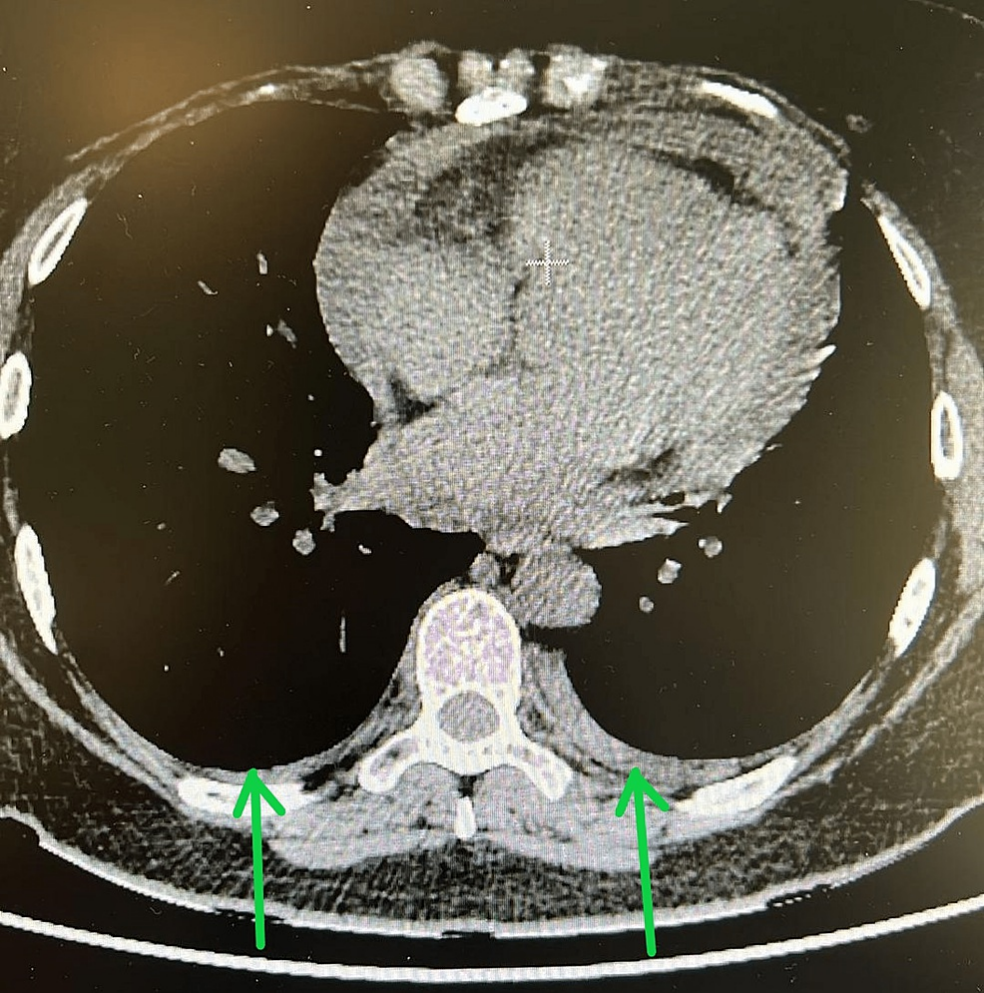
Chest angio-tomography with pleural effusion. Green arrows point to a slight bilateral pleural effusion.

The patient also underwent chest angio-tomography, which excluded the existence of pulmonary thromboembolism as the cause of dyspnea and chest pain and revealed a slight pericardial effusion (Figure 2) associated with thickening of the leaflets and a slight bilateral pleural effusion without changes in the lung parenchyma (Figure 3).

The diagnosis of myopericarditis was admitted at the entrance and the patient initiated therapy with acetylsalicylic acid (ASA) 500 mg three times per day and colchicine, with proposed hospitalization for the etiological study of this condition. 

On the first day in the hospital, a transthoracic echocardiogram was performed, which revealed the presence of a non-dilated left ventricle, without alterations in segmental kinetics and with good global systolic function, without valvular and vena cava alterations and small circumferential pericardial effusion without hemodynamic compromise.

Knowing that the main cause of myopericarditis in young patients without cardiovascular risk factors is a viral infection, often with slight symptoms, viral serologies were requested, namely, influenza A and B, enterovirus, cytomegalovirus and Epstein-Barr virus, herpes virus 1 and 2, parvovirus, hepatitis C and B virus, and human immunodeficiency virus. All returned negative. Although the patient denied contact with animals, recent trips and lives in an urban area, the investigation of serologies for bacterial infections with potential cardiac involvement revealed the presence of phase II antibodies IgM and IgG positive for *C. burnetti*, in the titles of 1:64 of IgM and 1:96 IgG raising the hypothesis of Q fever as the cause for this cardiovascular involvement. 

Taking into account the concomitant personal history of xerostomia, xerophthalmia and malar eczema, the study of autoimmunity was requested, which revealed the presence of a rheumatoid factor elevated to 10 times the upper limit of normal, sedimentation rate (SR) 105 mm/h, ANA antibodies positive at >1:80 titer, positive anti-dsDNA antibodies, positive anti-SSA antibodies with negative anti-SSB, and weakly positive lupus anticoagulant. Due to the high suspicion of being a case of SLE and secondary SS as a possible cause for this clinical picture, an ultrasound of the salivary glands was performed, which showed diffuse atrophy of the parotid and submaxillary glands, with the heterogeneous structure without nodular expression, image compatible with this same diagnosis. 

After all the etiological investigation, it was concluded that this is a case of myopericarditis and its cause would be related to the presence of an autoimmune disease not yet diagnosed at the time of the episode: simultaneous diagnosis of SLE and SS, raising the doubt whether the presence of positivity for the IgM and IgG antibodies of phase II for *C. burnetti* could mean the contribution of an eventual Q fever in the period of seroconversion as a cause for the cardiac involvement or even for the appearance of inaugural SLE flare. 

In this sense and after the diagnosis of SLE and SS, the patient started corticosteroid therapy with oral prednisolone 40 mg per day and hydroxychloroquine 200 mg per day, maintaining the initial medication for myopericarditis (ASA and colchicine). The patient maintained an indication to apply ocular lubricant daily and frequently throughout 24 h. During hospitalization, the patient registered a controlled arterial blood pressure profile and heart rates, with complete resolution of complaints of angor and fever, and normalization of both troponin and CRP values, so it was decided not to start doxycycline antibiotics until the serologies for *C. burnetti* were repeated at least four weeks after hospital discharge.

Before discharge, the patient was observed by Cardiology with the recommendation for maintaining therapy with ASA and colchicine for at least six months. Ophthalmology was also contacted to discuss the case, which was considered pertinent to reassess the patient in an appointment within one to two months after discharge under treatment for SLE and SS. 

After hospital discharge, the patient maintained follow-up in Internal Medicine appointments in order to monitor not only the evolution of the cardiac condition but also to control the autoimmune disease, repeating the autoantibody assays in a period of 12 weeks and the serologies for *C. burnetti* four weeks after discharge. In fact, after this period, the patient repeated blood tests which revealed positivity for ANA, anti-dsDNA, and anti-SSA. In the serologies for *C. burnetti*, the IgM phase II antibodies were negative this time, with an increase in the titer of the IgG antibodies, which is an indication that this is more likely to be a seroconversion of a past bacterial infection, rather than a finding by cross-reaction.

After discharge, the patient was instructed to follow a prednisolone weaning schedule, but to maintain chronically hydroxychloroquine therapy, which is indicated for both SLE and SS, in addition to maintaining ASA and colchicine until a new reassessment by Cardiology in six months. 

## Discussion

After the entire investigation that was carried out during hospitalization, we concluded that we were facing myopericarditis with a very particular and infrequent etiology. In fact, the patient showed up at the emergency department with lipothymia and angor accompanied by dyspnea and worsening tiredness for small efforts, with evidence of myocardial injury in the blood tests (increased values of a highly sensitive troponin I) that could suggest an acute coronary syndrome. However, in addition to these symptoms, the patient was febrile and had an increase in inflammatory/infection parameters at admission (leukocytosis and increased CRP). This, associated with the fact that she was a female young patient without apparent cardiovascular risk factors, it is important to think about other causes of angor as more likely, with myopericarditis as the first diagnostic hypothesis to investigate. 

This was followed by a set of complementary exams that corroborated this diagnosis: an electrocardiogram with unspecific changes in repolarization and a transthoracic echocardiogram without changes in myocardial segmental kinetics, with good systolic and diastolic function and with a small pericardial effusion. She started therapy for myopericarditis, with ASA and colchicine, and a whole process of investigating the underlying cause began. 

Since infection is the main cause of myopericarditis, serologies for viruses and bacteria were requested. However, in the case of a female patient, with a previous history of xerophthalmia and xerostomia that she devalued for five years, the search for autoantibodies directed to autoimmune diseases that are more directly related to sicca symptoms is simultaneously urgent. 

From all this research, it was possible to ascertain the presence of phase II IgG and IgM antibodies positive for *C. burnetti*. Q fever is a zoonosis and its agent *C. burnetti* has its main reservoir in animals such as sheep, goats, and pigeons. Vogiatzis et al. 2008 [5] realized that after aerosolized from biological fluids, it survives for long periods and can be transported by air over long distances, infecting animals or humans that did not necessarily have direct contact with the infected animal. This fact could justify the infection in this patient who denied contact with any type of animals and, therefore, did not present an epidemiological history suggestive of zoonosis. The diagnosis is serological, and the measurement of IgG and IgM antibodies should be done at the time of the appearance of the first symptoms and three to four weeks after the first determination. Phase I anti-*C. burnetti* antibodies (which are positive for IgG titers greater than 1: 800 in chronic infection) and phase II are the ones that are most frequently positive in the acute phase (with IgG with titers greater than or equal to 1: 200; or IgM greater than or equal to 1:50; or both) [5-6]. Thus, we have titers of phase II antibodies that satisfy the Q fever diagnosis criteria.

However, from the study of autoimmunity, it was found the presence of a significantly increased sedimentation rate and rheumatoid factor, as well as positivity for ANA, anti-dsDNA, and anti-SSA antibodies. 

The classification criteria for SLE were developed in 1971 and revised again in 1982 and 1997. However, the American College of Rheumatology developed, in 2019, classification criteria to be applied in published studies, in order to achieve high specificity and high sensitivity simultaneously. Validation cohorts suggested this goal was reached with a specificity of 93% and a sensitivity of 96% [3]. In 2022, Ameer et al. [3] cited the classification criteria for SLE, which include a positive ANA test followed by additive weighted criteria that are grouped into seven clinical (constitutional, hematological, musculoskeletal, mucocutaneous, serosal, renal, neuropsychiatric) and three immunological (SLE-specific antibodies, anti-phospholipid antibodies, complement proteins) domains. Patients with more than 10 points are classified as having SLE.

Based on these criteria, it was possible to make the definitive diagnosis of SLE in this clinical case with a total of 13 points, taking into account that we were in the presence of positivity for ANA, serositis (pleural and pericardial effusions, corresponding to five points), positive anti-dsDNA (corresponding to 6 points), and positive lupus anticoagulant antibodies (corresponding to two points).

Thomas et al. 2017 [7] conducted a multicentric study of 29 patients and reported that myocarditis is the inaugural manifestation that allows the diagnosis of SLE in 60% of patients. According to Tanwani et al. 2018 [8] even in already diagnosed SLE, myocarditis appears in 43% of patients aged young (average 33 years) in the initial course of the disease (first three years). 

The diagnosis of SLE is accompanied by a diagnosis of SS in 14%-17.8% of patients, a phenomenon called by some authors as polyautoimmunity [2]. SS is an autoimmune inflammatory disease that involves the destruction of the lacrimal and salivary glands leading to the consequent xerostomia and xerophthalmia condition lasting more than three months (dry eye and dry mouth symptoms) [9]. The main risk factors for the diagnosis of SS consist of the age of over 25 at the time of the initial diagnosis of SLE, female gender, and the presence of positive anti-SSA antibodies, all of which are present in this patient. 

In patients with both diagnoses, symptoms of sicca kerato-conjunctivitis are generally more severe with greater glandular destruction, a higher prevalence of periodontal disease and oral candidiasis, and a higher frequency of associated thyroiditis. They also have more prolonged flares and multiorgan involvement (osteoarticular, cardiovascular, and pulmonary) more frequent but with lower associated mortality [2].

There are several reports in the literature of clinical cases with the most different presentations, most even with myocarditis, pericarditis or both, in which an initial diagnosis of SLE is made, treatment for it is initiated and it is due to a therapeutic failure that new researches on etiology begin. In some cases, positivity in the serologies for *C. burnetti* is found as a confounding factor. Infection with this microorganism leads to an inflammatory process with the production of autoantibodies, so despite the fact that patients meet the criteria for the diagnosis of SLE, the improvement only occurs when initiated therapy with directed antibiotics (in almost all studies with doxycycline 100 mg two times per day).

It should be noted that this was not the case for our patient: she started immunosuppressive therapy (high dose corticosteroid and hydroxychloroquine), with a rapid satisfactory response, with complete resolution of symptoms and consistent analytical improvement without the need for antibiotic therapy. This leads us to think that, even though we have positivity in the phase II serologies for *C. burnetti* (dosed in low titers), this should not be the direct cause of myopericarditis but the main factor that originated the inaugural flare of SLE. Ohguchi et al. 2006 [10] described one case in the literature in which, in particular, *C. burnetti* infection may have been the cause of a destabilization of the immune system that led to the onset of an inaugural flare of SLE. It seems to us to be the same conclusion that we achieved from our patient's study: a possibly subclinical infection by *C. burnetti* as the cause for the appearance of a first inaugural flare of SLE, in a patient already with probable autoimmune conditions (already with complaints compatible with SS never investigated in the past and therefore without immunosuppressive therapy instituted to control the disease). 

## Conclusions

In the Internal Medicine re-evaluation appointment four weeks after hospital discharge, she was asymptomatic, with apparently controlled disease and with positive phase II serologies for *C. burnetti*, which again were suggestive of past infection already solved without chronicity (she kept phase I negative antibodies). At the end of 12 weeks after the initial diagnosis, she maintained positive ANA antibodies and anti-dsDNA and anti-SSA antibodies, which confirms once again the hypothesis that we defend here: diagnosis of myopericarditis as a presentation of the inaugural flare of SLE triggered by *C. burnetti* infection.

With this case report, we should always remember that typical angor in a young patient without cardiovascular risk factors should raise the clinical suspicion of an inflammatory condition of myocarditis/pericarditis and not necessarily an acute coronary syndrome as the first diagnostic hypothesis. Infections are often confounding factors for the diagnosis of autoimmune diseases due to the production of autoantibodies by our immune system in the acute phase of infection, but the early distinction between infectious vs. autoimmune etiologies is very important in order to establish the appropriate therapy.
